# A High-Throughput Approach for Identification of Novel General Anesthetics

**DOI:** 10.1371/journal.pone.0007150

**Published:** 2009-09-24

**Authors:** Wendy A. Lea, Jin Xi, Ajit Jadhav, Louis Lu, Christopher P. Austin, Anton Simeonov, Roderic G. Eckenhoff

**Affiliations:** 1 NIH Chemical Genomics Center, National Human Genome Research Institute, National Institutes of Health, Bethesda, Maryland, United States of America; 2 Department. of Anesthesiology & Critical Care, University of Pennsylvania School of Medicine, Philadelphia, Pennsylvania, United States of America; Griffith University, Australia

## Abstract

Anesthetic development has been a largely empirical process. Recently, we described a GABAergic mimetic model system for anesthetic binding, based on apoferritin and an environment-sensitive fluorescent probe. Here, a competition assay based on 1-aminoanthracene and apoferritin has been taken to a high throughput screening level, and validated using the LOPAC^1280^ library of drug-like compounds. A raw hit rate of ∼15% was reduced through the use of computational filters to yield an overall hit rate of ∼1%. These hits were validated using isothermal titration calorimetry. The success of this initial screen and computational triage provides feasibility to undergo a large scale campaign to discover novel general anesthetics.

## Introduction

General anesthetics are used so commonly today that it is difficult to escape life without having been exposed to them. Despite their widespread use, no new general anesthetics have been developed for over 30 years; all current development seems targeted at pharmacokinetics as opposed to pharmacodynamics. But this is not because these drugs have been optimized in terms of specificity and side effect profile. Indeed, there is growing concern that general anesthetics, especially the volatile ones, are associated with cognitive effects that long outlast their residence in the brain [1]. Thus, a need exists for new general anesthetics with improved safety and specificity.

Previous development of general anesthetic drugs has always been empirical, or based on non-specific physicochemical properties, such as hydrophobicity. This is a result of not having validated protein targets, or not having high resolution structures of even putative targets, such as the GABA_A_ receptor [2]. We have recently reported that a soluble protein, apoferritin, mimics the pharmacodynamic behavior of general anesthetic targets, and more specifically the GABA_A_ receptor [3], [4]. Further, this protein is readily crystallized and x-ray diffraction data of the anesthetic protein complex resolved to high resolution [5]. This apoferritin site binds specifically a wide range of general anesthetics, including those that are inhaled and those that are injectable, and excludes the non-immobilizers [6]. Therefore, we reasoned that this site might serve as a platform for the first protein-based anesthetic screening effort.

Screening efforts require a robust assay to report on binding or an activity change in the target. Since our previous work with apoferritin did not identify significant changes in apoferritin activity on occupancy of the anesthetic site, we sought an assay to report on occupancy alone. Most such assays employ fluorescence competition, whereby a fluorescent reporter molecule is displaced by compounds that also bind the site. A suitable candidate was identified, and the binding and fluorescence properties of 1-aminoanthracene (1-AMA) have recently been reported [3]. Further, we have shown that known general anesthetics (e.g., isoflurane and propofol) inhibit 1-AMA fluorescence (binding) with IC_50_ values that closely approximates their K_D_ obtained through an independent method (isothermal titration calorimetry) [3]. In this communication, we report on the miniaturization of this assay and its validation in high throughput screening mode using the LOPAC^1280^ library of bioactive molecules.

## Results

### Assay Miniaturization

**Figure 1:**
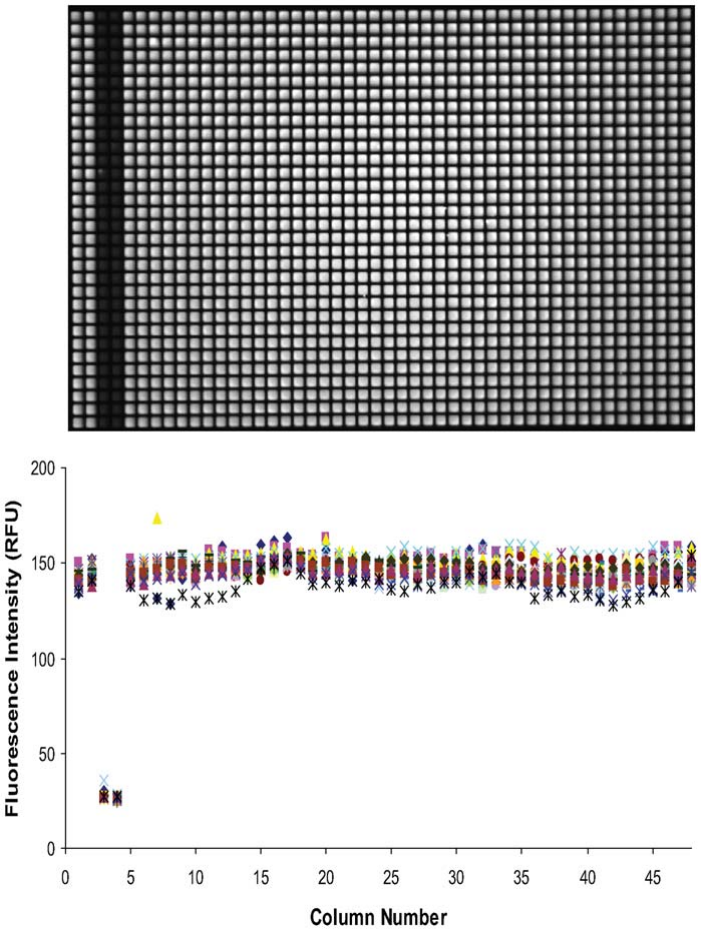
Assay Miniaturization to 1,536-well format. Plate image (above) is shown with quantitation (below). Columns 1,2,5–48 are 15 µM ApoF + 1-AMA, column 3,4 are free 1-AMA. 3 µL total volume, 15 min. incubation prior to fluorescence read. Top  =  raw ViewLux CCD image; bottom  =  RFU data from CCD image demonstrating consistency. The Z' factor and signal-to-background calculated from the first 128 wells of data (left four columns) were 0.87 and 5.3, respectively.

**Figure 2:**
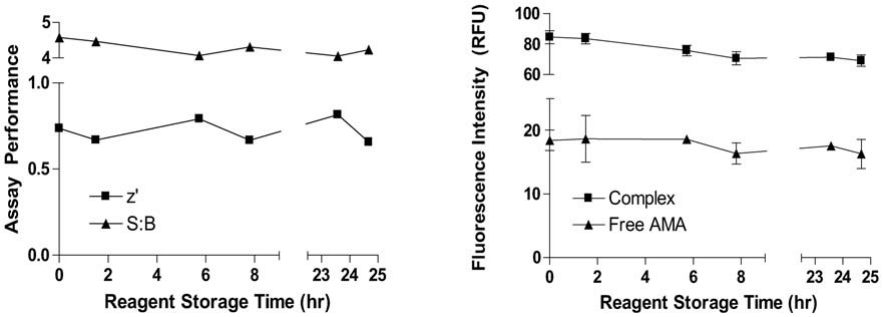
Assay stability. Bottles with free 1-AMA or complex were stored at 4°C, connected to a liquid dispenser at shown time points, and the assay performed as described above. Fluorescence intensity, signal-to-background ratios (S∶B) and Z' factor were computed from 64 column 1,2 wells (complex) and 64 column 3,4 wells (free 1-AMA).

The previously-reported apoferritin-1-AMA binding assay was miniaturized to 3 µL in 1,536-well plates. Baseline plate reads with no added compound demonstrated robust signal and excellent well-to-well uniformity in 1,536-well format (Figure 1). When 50% saturated 1-AMA was complexed with 15 µM apoferritin, the fluorescence increased 5.3-fold relative to free 1-AMA and the associated Z' factor [7] exceeded 0.85 (Figure 1). This result was reproduced with two lots of horse-spleen apoferritin and upon repeated testing. Robust signal was maintained when the apoferritin concentration was lowered to 8 µM, in order to lower the protein consumption. Figure 2 also demonstrates that the assay reagents, as formulated at their screening concentrations, were stable for over 24 hours: both the Z' factor and the signal-to-background ratio remained flat for the duration of the stability test. This excellent overnight stability coupled with robust assay performance in 1,536-well plate format indicates that the assay can be screened in an automated and unattended fashion.

### Quantitative High Throughput Screening (qHTS) of LOPAC^1280^ Library

**Figure 3:**
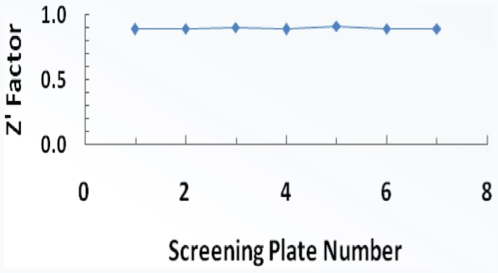
Robustness of screening assay: Z' factor during the experiment averaged 0.89.

**Figure 4:**
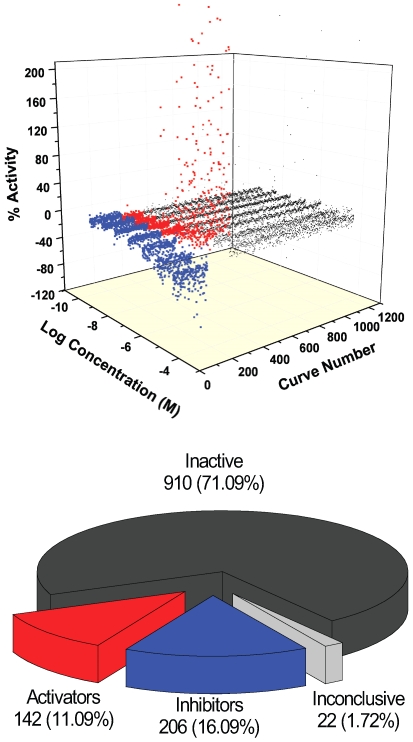
Cumulative results of the screen. Compounds are grouped according to inhibitor (blue), activator (red), inactive (dark grey) and inconclusive (light grey) categories.

The LOPAC^1280^ library was screened in qHTS mode [8] using the above described 1-AMA/apoferritin assay, with library compounds tested at seven concentrations in the range of 77 µM to 25 nM. The assay performance remained robust over the course of the screen, with high Z' factor maintained throughout (Figure 3). Detailed results are provided in PubChem (PubChem AID to be provided upon manuscript acceptance). Figure 4 shows the cumulative library response in both 3D and pie format, representing the activity distribution of compounds. Concentration response curves (CRCs) were categorized into three groups: inactive, active, inconclusive based on the quality of the CRC and the maximum response of the compound. First, inactive compounds have a maximum response of less than 3 sigma of the assay (10% inhibition for the LOPAC^1280^ screen). The qHTS yielded 910 compounds classified as inactive. Additionally, 142 of the compounds displayed a signal increase in the assay. These potential ‘activators’ were considered artifacts and were also categorized as inactives. Among the compounds that produced a signal decrease, CRCs with a partial or full response and a maximum response greater than 60% inhibition were categorized as active (5 compounds). An additional 201 compounds had weaker inhibitory response, where the CRC r^2^ was less than 0.9 (noisy curves) or where the maximum response was less than 6 sigma. These less reliable responses were nevertheless carried through the next steps of triaging together with the 5 actives. Finally, a small set of compounds (22) had curves that were unclassified. These were noisy responses that showed some signal change only at the highest concentration tested and were thus considered to be inactive (listed as inconclusive in the pie chart of Figure 4).

### Filtering of Screening Hits

Most of the 206 inhibitors yielded incomplete CRCs. Physicochemical filtering of the 206 compounds eliminated all but 21 compounds. It is assumed that qHTS signal inhibition in the eliminated compounds was largely the result of inner-filter or aggregation effects. Finally, compounds that were classified as “inhibitors” based only on a single point of activity were eliminated (2/21 compounds) to yield a final list of 19 active compounds.

### ITC

**Figure 5:**
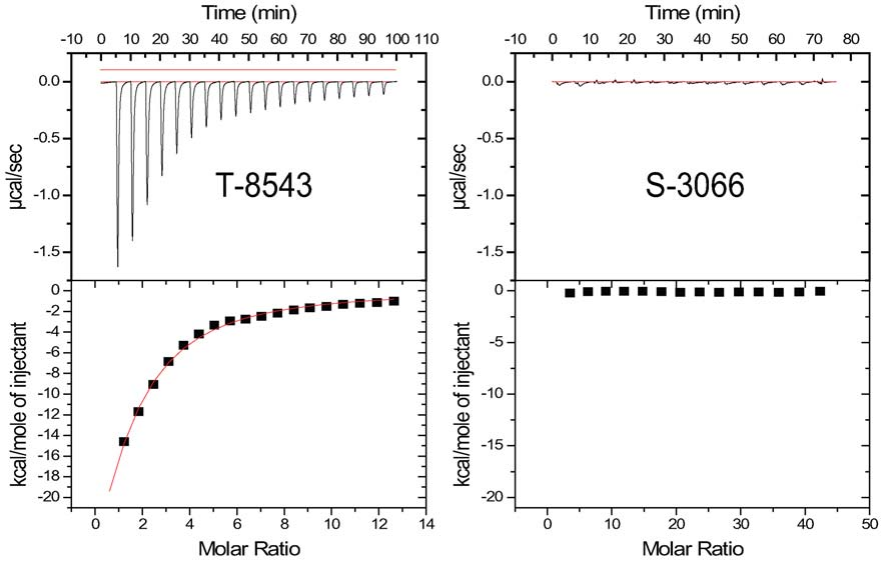
Typical ITC enthalpograms. LOPAC compound T-8543 shows clear exothermic binding behavior (A), while S-3066 does not (B).

**Figure 6:**
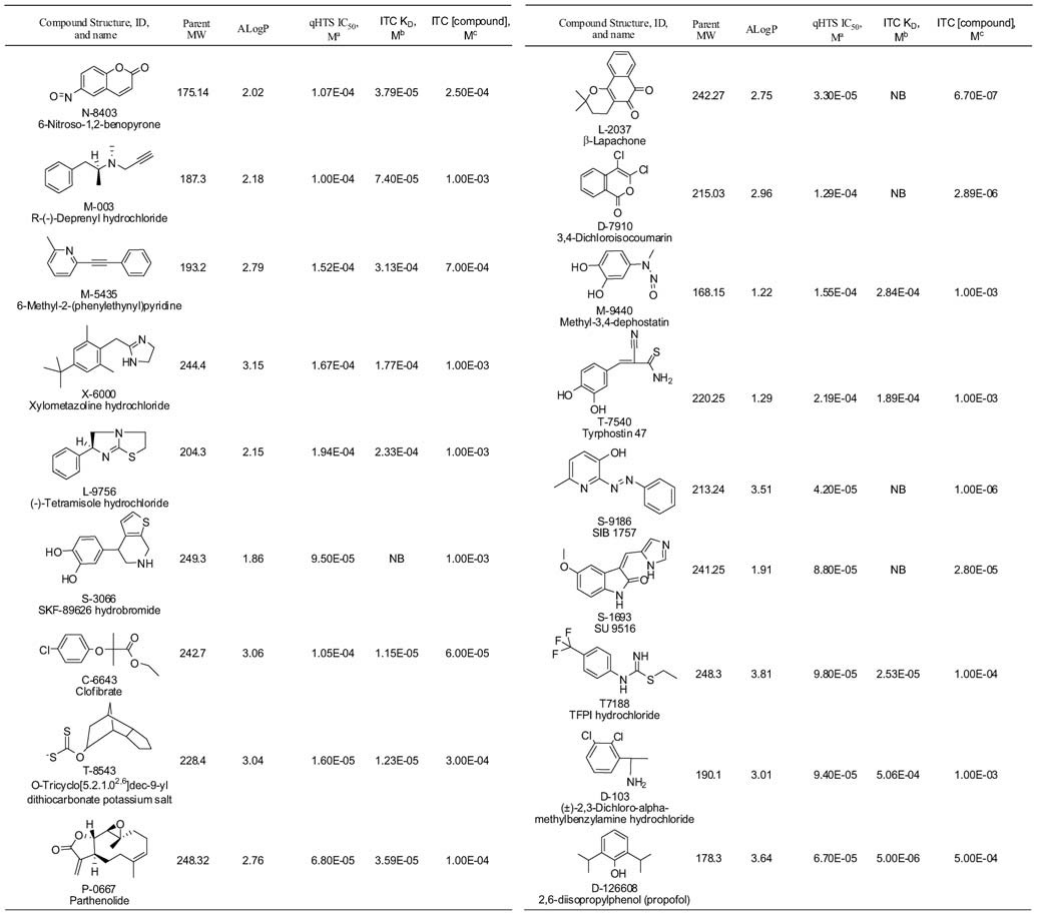
Final compound characteristics and binding summary. Shown are the eighteen compounds, their stick structures and chemical names. The qHTS IC_50_ is computed directly from the 1-AMA inhibition data, in molar units, fixing the infinite value to −70%. The ITC K_D_ is derived from single class fits to enthalpograms (n = 1), and is in molar units (NB  =  no binding). The ITC [compound] column represents the molar concentration of compound achieved after mixing and filtration prior to loading into ITC syringe. Target concentration was 1 mM in each case, but clearly not achieved in all. Compound concentrations were measured with UV absorbance.

**Figure 7:**
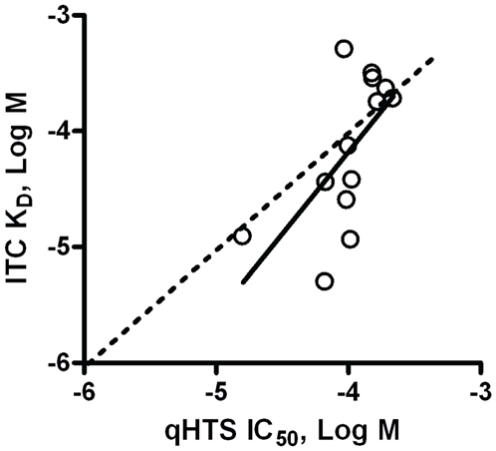
Relationship between qHTS IC_50_ and ITC K_D_. Shown are 13 compounds for which ITC experiments provided reliable parameters, together with the fit (solid line, R^2^ = 0.4; P = 0.02) and the line of identity (dotted).

Eighteen of the 19 compounds could be obtained from the primary suppliers for the low-throughput validation study. Of the 18 compounds, 11 yielded unambiguous evidence of a classic exothermic binding interaction (see Figure 5A for an example), and two provided endothermic relationships. Of the five giving ambiguous data, or no heat signal at all (see Figure 5B for an example), low compound solubility appears to be the dominant reason. Figure 6 gives a list and structure of all the obtained compounds, their physical parameters, and the results from both qHTS and ITC. Considerable noise exists in the relationship between the IC_50_ calculated from qHTS data and the K_D_ calculated from the enthalpograms (Figure 7), presumably because the high protein (8 µM) and probe (15 µM) concentrations, and limitations to compound solubility, produce incomplete curves in the qHTS experiment. Nevertheless, the relationship between qHTS and ITC measures of affinity is significant, and does not deviate significantly from the line of identity.

## Discussion

Despite decades of study, the mechanism of anesthetic action has not emerged, and as a result, design of new anesthetic drugs has been reduced to empiricism. There is general agreement, however, that direct interactions with protein underlie the desirable effects of anesthetics, lending hope that novel compounds can be found. The problem has been the lack of validated, structurally accessible targets on which to focus. The GABA_A_ receptor/ion channel, for example, is thought by many investigators to be an important anesthetic target [9], yet only homology models based on a low resolution cryoEM structure of the nicotinic acetylcholine receptor are available [10]. Further, there is only a general idea of where the anesthetic site on this large heteroligomer resides [2], [11]. Thus, a surrogate approach, based on another protein that displays pharmacodynamic mimicry [4], [5], was employed here. The assay method is based on a fluorophore that binds the anesthetic cavity on apoferritin, shows a large increase in fluorescence on binding, and is a general, GABAergic anesthetic itself [3].

The number of raw hits obtained in this screen is large, but the filtering based on known cavity characteristics [4], [5], shows that most are due to interactions other than competitive binding at the anesthetic site. These interactions include fluorescence interference and aggregation, although the latter is expected to enhance signal rather than reduce because of partitioning of 1-AMA into the compound micelle; indeed, we observed 142 such compounds in the screen. Fluorescence interference will primarily be due to inner-filter effect, whereby the compound absorbs the excitation or emission wavelengths of 1-AMA. For example, compounds S-1693 and S-3066 were classified as inhibitors by qHTS, each producing ∼30% inhibition at maximal concentrations (77 µM), but ITC failed to provide convincing evidence of binding. This is likely due to considerable absorbance at the excitation wavelength (340 nm). Future assays will take advantage of the broad absorbance band of 1-AMA, and red-shift the excitation wavelength closer to 400 nm. This should also have the advantage of better signal to noise ratios, permitting decreased reagent consumption.

**Figure 8:**
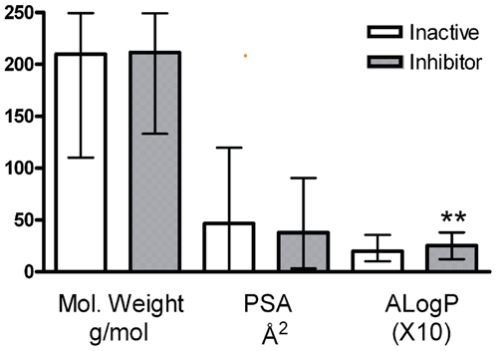
Filter performance. Applying screening criteria to both the inhibitor and inactive groups recovered 21/206 and 157/910 compounds respectively. In each of these filtered groups, the mean and range for molecular weight, polar surface area (PSA) and octanol/water partition coefficient (ALogP) are shown. While the inhibitor compounds are significantly more hydrophobic, as suggested by the higher mean ALogP (**, P<0.005), the overlap is large and illustrates that the filters alone provide insufficient discrimination.

The simple filters used here take advantage of the fact that apoferritin has a high (1.7 Å) resolution structure, the general anesthetic binding site has been identified, and it is an enclosed cavity, rather than a surface patch [5]. Thus, there are defined limitations on the size and physiochemical character of compounds which can be accommodated in this site. Further, because of the stability of the apoferritin oligomer, this site is unlikely to undergo significant “induced fit”. We chose filter parameters broad enough to capture novel compounds, but exclude those clearly outside the range that could be accommodated by the apoferritin site. We recognize that our filtering process is essentially a partial return to empiricism, but we note that it retains the important element of targeted protein binding. That the protein site is providing the dominant selectivity, as opposed to the filters, is illustrated by the fact that these filters alone select 176 of 910 *inactive* LOPAC compounds. Figure 8 shows that while there is the expected difference in mean hydrophobicity between screened inactive and inhibitors, the overlap is large. This suggests that the 3 dimensional interactions of compound atoms with those of the cavity lining, (e.g., the pharmacophore) have the largest influence on binding.

Inspection of the final, validated hits reveals no obvious pharmacophore, chemical group, or structural feature to explain binding. Final compounds range in size between 168 and 250 MW, have one to three rings, and a variety of bulky, generally hydrophobic constituents. But several have charged or polar groups, no doubt to aid solubility. It is important to realize that the LOPAC set already have validated drug activities and many are clinically used for this purpose. Only one general anesthetic is included in LOPAC, propofol, and it was successfully identified in this screen. Since general anesthesia is an important side effect that would be selected against in the development of other drugs, we did not predict many useful hits in this feasibility screen. Nevertheless, several candidate compounds were identified for further testing using receptor or *in vivo* assays. It is interesting to note that several compounds have documented activities shared by some of the existing general anesthetics: inhibition of glutamate, adrenergic and dopamine receptors, nitric oxide synthase, monoamine oxidase and various kinases and phosphatases [12]. It is possible that something like general anesthesia is elicited at concentrations of these compounds higher than needed to produce their current primary effects.

We validated the final list using isothermal titration calorimetry, an entirely different and low throughput methodology, to detect and characterize favorable interactions between two reactants (in this case, LOPAC compound and apoferritin). Most compounds showed unambiguous evidence of a favorable interaction, but several did not. We found that the majority of ITC-negative compounds were the least soluble, meaning that our saturated solution did not contain adequate concentrations of compound to achieve enough occupancy in the apoferritin site to permit detection at the high-protein ITC conditions. Worse, the ITC experiment only achieves a compound concentration of about 17% of the saturated concentration in the syringe (see Figure 6). Furthermore, the DMSO included in the qHTS assay may have increased compound concentration to the point where 1-AMA displacement could be observed. Such co-solvents are typically not included in ITC experiments because of extreme dilution heats if the buffers are not precisely matched. Further prosecution of such compounds is not warranted as very hydrophobic compounds tend to be poor anesthetics [6], a phenomenon generically related to the cut-off effect [13]. At the same time, more soluble compounds tend to be low affinity anesthetics, thus expanding the screen to increase compound concentrations (by delivering double the amount of compound via two successive pin transfers) is not considered a productive strategy.

In summary, we have developed and tested the feasibility of a quantitative high throughput assay of a general anesthetic protein binding site. This initial screen of the LOPAC^1280^ library of compounds demonstrates that the 1-AMA/apoferritin assay can be used in miniaturized high-throughput screening mode, and sets the stage for screens of large-size compound libraries. A high rate of false positives can be addressed through simple filters to achieve an overall hit rate of about 1% of a validated drug library. Refinements in screening methodology and post-screening triaging is expected to further limit the yield of false positives.

## Methods

### Materials

1-AMA and horse spleen apoferritin were obtained from Sigma and used without further purification. Dimethyl sulfoxide (DMSO, certified ACS grade) was from Fisher, Inc. The Library of Pharmacologically Active Compounds (LOPAC1280, Sigma-Aldrich) was received as a set of 10 mM DMSO solutions and formatted in 1,536-well compound plates as a dilution series [7]. Preliminary studies showed only a modest reduction in 1-AMA/apoferritin signal intensity (∼10%) at the maximal expected DMSO concentration of 1%. Medium binding black solid-bottom 1,536-well plates (assay plates), and 1,536-well polypropylene plates (compound plates) were purchased from Greiner Bio One (Monroe, NC).

### Screening

Three µL of reagents (free 1-AMA (50% saturated solution of 1-aminoanthracene in PBS) in columns 3,4 as negative control and 1-AMA/apoferritin mixture (10 µM apoferritin in 50% saturated 1-aminoanthracene in PBS (∼15 µM)) in columns 1, 2, 5–48) were dispensed into 1,536-well Greiner black assay plates. Compounds (23 nL) were transferred via Kalypsys pintool equipped with 1,536-pin array (10 nL slotted pins, V&P Scientific, San Diego, CA). The plates were incubated for 10 min at room temperature, and then read on a ViewLux high-throughput CCD imager (Perkin-Elmer, Waltham, MA) using standard UV excitation filter (340 nm, bandwidth 60 nm) and fluorescein emission filter (540 nm, bandwidth 25 nm). Throughout the screen, reagent bottles and all liquid lines were made light-tight to minimize reagent degradation. Activity was computed as the normalized fluorescence response relative to free 1-AMA and 1-AMA/apoferritin complex values. Concentration–effect relationships were derived by using publicly-available curve-fitting algorithms developed in-house (http://ncgc.nih.gov/pub/openhts/). A four parameter Hill equation was fitted to the concentration-response data by minimizing the residual error between the modeled and observed responses. Compounds were classified as either active inhibitor, active activator, inconclusive or inactive as described in the results.

### Data filtering

Compounds showing enhancement of signal intensity are assumed to represent either fluorescence interference or aggregation phenomena. Thus, for this assay, hits were defined as only those that showed inhibition of signal intensity, comprising complete or partial concentration response curves (CRCs). These hits were then further truncated through the use of two computational filters. Because the character of this deep pocket, with respect to size and physicochemical character, is well known [5], we eliminated any compounds larger than 250 Da, and any with ALogP values outside the range of 1–4. Clinically used general anesthetics are all consistent with these filters.

### Isothermal Titration Calorimetry (ITC)

The filtered compound list was then subjected to a secondary validation using a direct binding technology, isothermal titration calorimetry (ITC) (MicroCal VP-ITC, Northampton, MA). Compounds were obtained directly from Sigma, Altan, Tocris or Acros, and ∼1 mM solutions were prepared in a phosphate buffered saline (PBS). This was accomplished through vigorous shaking and sonication of the solution, followed by filtration through 0.2 µm PTFE syringe filters. Concentrations were then confirmed with absorption spectroscopy. Several compounds were found to be far less soluble in aqueous buffer than 1 mM, limiting the ability of ITC to determine binding (see below). The ITC cell was loaded with 2.5 mg/ml apoferritin, and the syringe loaded with the compound solution. Titrations were conducted at 20°C, subtracted by buffer to buffer and compound to buffer runs, and the enthalpograms fitted to a single class binding site model using Origin 7.0. Each compound was run in duplicate.

## References

[1] Baranov D, Bickler PE, Crosby GJ, Culley DJ, Eckenhoff MF (2009). Consensus statement: First International Workshop on Anesthetics and Alzheimer's disease.. Anesth Analg.

[2] Jurd R, Arras M, Lambert S, Drexler B, Siegwart R (2003). General anesthetic actions in vivo strongly attenuated by a point mutation in the GABA(A) receptor beta3 subunit.. FASEB J.

[3] Butts CA, Xi J, Brannigan G, Saad AA, Venkatachalan SP (2009). Identification of a fluorescent general anesthetic, 1-aminoanthracene.. Proc Natl Acad Sci U S A.

[4] Vedula LS, Brannigan G, Economou NJ, Xi J, Hall MA (2009). A unitary anesthetic-binding site at high resolution.. J Biol Chem.

[5] Liu R, Loll PJ, Eckenhoff RG (2005). Structural basis for high-affinity volatile anesthetic binding in a natural 4-helix bundle protein.. FASEB J.

[6] Koblin DD, Chortkoff BS, Laster MJ, Eger EI, Halsey MJ (1994). Polyhalogenated and perfluorinated compounds that disobey the Meyer-Overton hypothesis.. Anesth Analg.

[7] Zhang JH, Chung TD, Oldenburg KR (1999). A Simple Statistical Parameter for Use in Evaluation and Validation of High Throughput Screening Assays.. J Biomol Screen.

[8] Inglese J, Auld DS, Jadhav A, Johnson RL, Simeonov A (2006). Quantitative high-throughput screening: a titration-based approach that efficiently identifies biological activities in large chemical libraries.. Proc Natl Acad Sci U S A.

[9] Campagna JA, Miller KW, Forman SA (2003). Mechanisms of actions of inhaled anesthetics.. N Engl J Med.

[10] Mascia MP, Trudell JR, Harris RA (2000). Specific binding sites for alcohols and anesthetics on ligand-gated ion channels.. Proc Natl Acad Sci USA.

[11] Li GD, Chiara DC, Sawyer GW, Husain SS, Olsen RW (2006). Identification of a GABAA receptor anesthetic binding site at subunit interfaces by photolabeling with an etomidate analog.. J Neurosci.

[12] Moody E, Skolnick P (2001) Molecular Bases of Anesthesia..

[13] Eckenhoff RG, Tanner JW, Johansson JS (1999). Steric hindrance is not required for n-alkanol cutoff in soluble proteins.. Mol Pharmacol.

